# On How Network Architecture Determines the Dominant Patterns of Spontaneous Neural Activity

**DOI:** 10.1371/journal.pone.0002148

**Published:** 2008-05-14

**Authors:** Roberto F. Galán

**Affiliations:** Department of Neurosciences, School of Medicine, Case Western Reserve University, Cleveland, Ohio, United States of America; Indiana University, United States of America

## Abstract

In the absence of sensory stimulation, neocortical circuits display complex patterns of neural activity. These patterns are thought to reflect relevant properties of the network, including anatomical features like its modularity. It is also assumed that the synaptic connections of the network constrain the repertoire of emergent, spontaneous patterns. Although the link between network architecture and network activity has been extensively investigated in the last few years from different perspectives, our understanding of the relationship between the network connectivity and the structure of its spontaneous activity is still incomplete. Using a general mathematical model of neural dynamics we have studied the link between spontaneous activity and the underlying network architecture. In particular, here we show mathematically how the synaptic connections between neurons determine the repertoire of spatial patterns displayed in the spontaneous activity. To test our theoretical result, we have also used the model to simulate spontaneous activity of a neural network, whose architecture is inspired by the patchy organization of horizontal connections between cortical columns in the neocortex of primates and other mammals. The dominant spatial patterns of the spontaneous activity, calculated as its principal components, coincide remarkably well with those patterns predicted from the network connectivity using our theory. The equivalence between the concept of dominant pattern and the concept of attractor of the network dynamics is also demonstrated. This in turn suggests new ways of investigating encoding and storage capabilities of neural networks.

## Introduction

A major challenge in current neuroscience is to understand the emergence of coherent complex activity from the interactions between neurons and its role in normal and pathological brain function. Approaches to facing this challenge have become more urgent in the last few years, as experimental techniques to record from many neurons simultaneously are being developed and improved, providing valuable data sets for analysis [1], [2]. These techniques have revealed that, even in the absence of stimulation, network activity organizes in complex spatiotemporal patterns [1], [3]–[8] that reflect, at least to some extent, the underlying network architecture [5], [6]. Likewise, recent experimental studies *in vitro* and *in vivo* have shown that cortical networks tend to reproduce spontaneous patterns consistently, known as cortical songs [8] because of their reliable temporal modulation. Although complementary studies suggest that these motifs are fully arbitrary [9], parallel studies of propagation of up-and-down states have shown highly stereotypical motifs in cortical circuits locally, leaving open the functional role of the cortical song and spontaneous activity in the brain [10]. Together, these results vindicate the necessity to understand in detail how neural circuitry constrains the repertoire of activity patterns that a network supports. This goal turns out to be even more significant, as we realize that encoding capabilities and storage capacity in a neural network are likely to rely on those repertoires. In this paper, we make a step toward this goal by showing both mathematically and in computer simulations how network connectivity determines the dominant patterns, or *modes* of the spontaneous activity.

We are focusing here on a rather microscopic level, where only the interactions among few cortical columns are investigated. Several authors have extensively studied the link between network architecture and network activity at the level of pathways and connections between brain areas previously [11]–[16]. Whereas the philosophy and mathematical framework used in this article parallel with those of the aforementioned studies, here we concentrate on the spontaneous activity: in addition to showing the link between network architecture and spontaneous activity, we also demonstrate the equivalence between dominant modes and network attractors.

## Results

**Figure 1:**
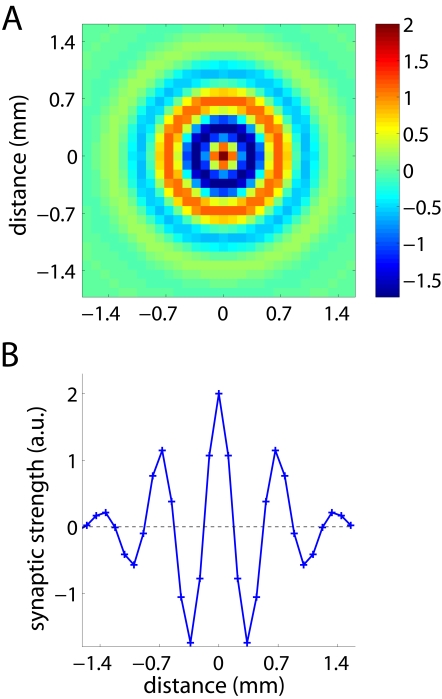
Biologically inspired connectivity. A: Synaptic strengths of an arbitrary neuron located at the center with its neighbors as a function of distance in two dimensions (Gabor kernel). Positive values indicate excitatory connections and negative values indicate inhibitory connections. B: Projection of the synaptic kernel along an axis crossing the center. Excitatory and inhibitory synapses are spatially periodic, interleaved and their strength decays with distance.

Using the general model of neural network dynamics described in *Materials and Methods*, we have simulated spontaneous activity in a neural network whose architecture resembles the patchy structure of horizontal connections in the neocortex of macaques and other mammals [17]. In particular, the sign and strength of synaptic connections between a given neuron and the rest of neurons in the network are approximated by a Gabor function (Fig. 1A and 1B). Other authors have previously used this synaptic kernel to model network dynamics in the prefrontal cortex during working memory tasks [18]. Nonetheless, the analyses below yielded qualitatively the same results with a Mexican-hat and with a Gabor kernel. While the vast literature on synaptic connections makes this a reduced model, our purpose here is to focus on a plausible architecture that allows us to illustrate the accuracy of our theoretical predictions on the relationship between anatomical connectivity and the patterns of spontaneous activity.

**Figure 2:**
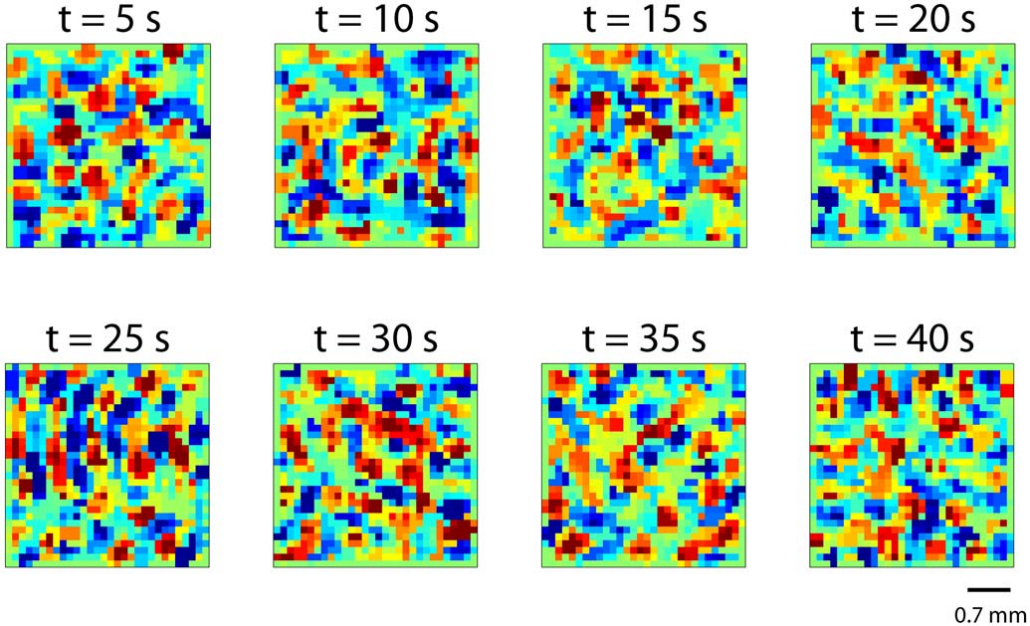
Snapshots of spontaneous activity. The patterns of spontaneous activity display excited (red) and inhibited (blue) spots with respect to the baseline firing rate (green) that evolve in time (red spots can turn blue and vice versa). The whole movie of the spontaneous activity is provided as Movie S1 in *Supporting Information*.

In the absence of stimulation, our network is driven by intrinsic, random background fluctuations modeled as uncorrelated, white noise; noise which emerges from fluctuations in channel opening and closing events and spontaneous synaptic release, among other sources of biological variability. These random currents trigger moderate firing rates in single neurons that propagate through the network as a result of synaptic interactions. This form of spontaneous activity organizes in complex spatiotemporal patterns. An example of simulated spontaneous activity is provided as Movie S1 of the *Supporting Information*. Some snapshots of the movie are also shown in figure 2. The color scale indicates the spontaneous firing rates in arbitrary units: red being above firing baseline (represented in green) and blue below. A significant feature of these snapshots is that they reveal a striking modularity, reminiscent of the spatial patterns observed via voltage sensitive dye imaging in V1 of cats [5]. In effect, red and blue areas segregate forming domains of approximately the same extension. These modules vary in time but have a pronounced tendency to reemerge, as shown in the movie.

**Figure 3:**
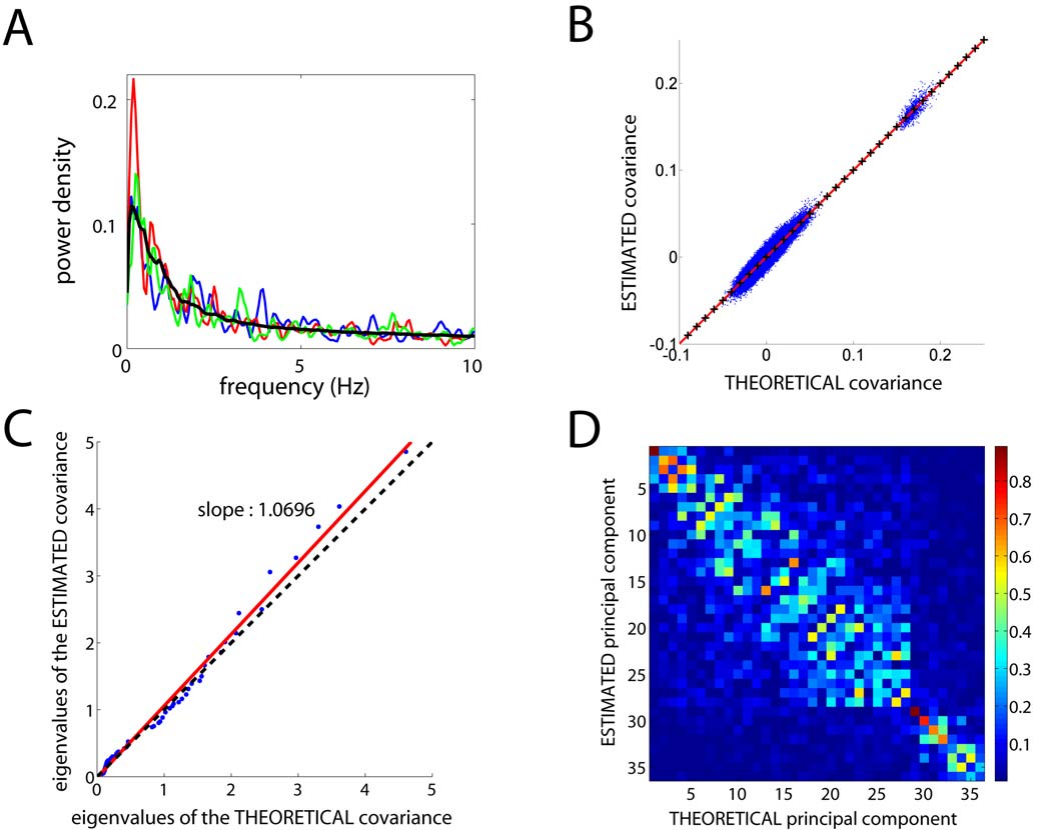
Properties of the spontaneous activity. A: Power spectral density of the arbitrarily chosen neural traces (blue, red, green) and the average across all neural traces (black). Neurons have some oscillatory behavior in the low frequency band (<5 Hz). B: The elements of the theoretically predicted covariance matrix and of the estimated covariance matrix coincide remarkable well (blue dots), as shown by a linear regression (red line) that perfectly overlaps with the identity (*y* = *x*, black crosses). C: The eigenvalues of both matrices (blue dots) are accordingly highly correlated (regression in red; identitiy in black dashed lines). D: The principal components of both matrices are also highly correlated. Note the pronounced band along the diagonal of their cross-correlation matrix, which indicates high similarity of the predicted and the estimated dominant modes.

Figure 3A shows the power spectral density of traces from three arbitrarily chosen neurons as well as the average across all neurons. Single neurons clearly have some oscillatory components revealed by peaks in the spectral density. However, not all the network is oscillating with the same frequency as indicated by different positions of the peaks for different neurons. In fact, on average there is no preferred frequency (Fig. 3A, black line). As explained below, the spontaneous activity consists of a summation of network modes analogue to the vibrations of a drum's membrane, which can be represented as the superposition of two-dimensional modes (Bessel functions) that oscillate in time at different frequencies. In the neural network, the spatial modes also oscillate at different frequencies, and a given neuron participates in many of these modes but with different weights. Therefore, the spectral densities of different neural traces are typically different as well.

As a first step to uncover the dominant patterns of the simulated spontaneous activity we calculate the covariance matrix from the spontaneous activity, which consists of the products of each pair of neural traces (pixels of the movie) averaged in time (see *Materials and Methods*). We then compare the covariance matrix of the spontaneous activity from the simulations with the covariance matrix derived analytically from the connectivity matrix in the mathematical model (see *Materials and Methods*). Remarkably, a strong correlation can be seen between the elements of both matrices (Fig. 3B). Then, we compute the dominant patterns of the spontaneous activity as the principal components, i.e. as the eigenvectors of the covariance matrix. We also compute their relative weight, i.e. the associated eigenvalues. When we do this from the covariance matrix of the simulated data and from the covariance matrix derived mathematically from the connectivity matrix (see *Materials and Methods*), we obtain a remarkable agreement (Fig. 3C and D). Note the pronounced band along the diagonal of the in Fig. 3D, which indicates high similarity (cross-correlation) between the predicted and the estimated dominant modes.

**Figure 4:**
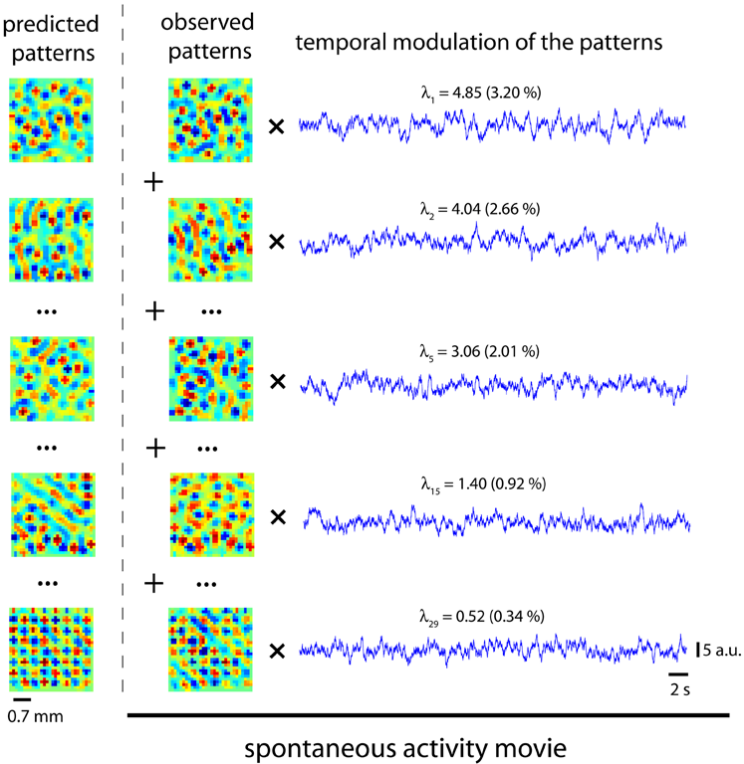
Decomposition of the spontaneous activity in dominant modes. The spontaneous activity can be mathematically described as a linear superposition of spatial modes (principal components) modulated in time. On the left, we compare some predicted spatial modes with the observed ones noting a good agreement overall. The blue traces on the right represent the temporal modulation of each pattern. The eigenvalue associated with the *i*-th principal component, or equivalently, the mean quadratic amplitude (variance) of that mode in the spontaneous activity is given by λ*_i_*. The relative variance contained in that mode is expressed as a percentage in parentheses.

In figure 4, the dominant patterns predicted by our theory are compared with the dominant patterns estimated from the simulations. Again, we note a good agreement, especially in the size and distribution of interleaved spots of excitation and inhibition. These results demonstrate that our theory can predict the dominant modes of the spontaneous activity just by knowing the architecture of the network.

**Figure 5:**
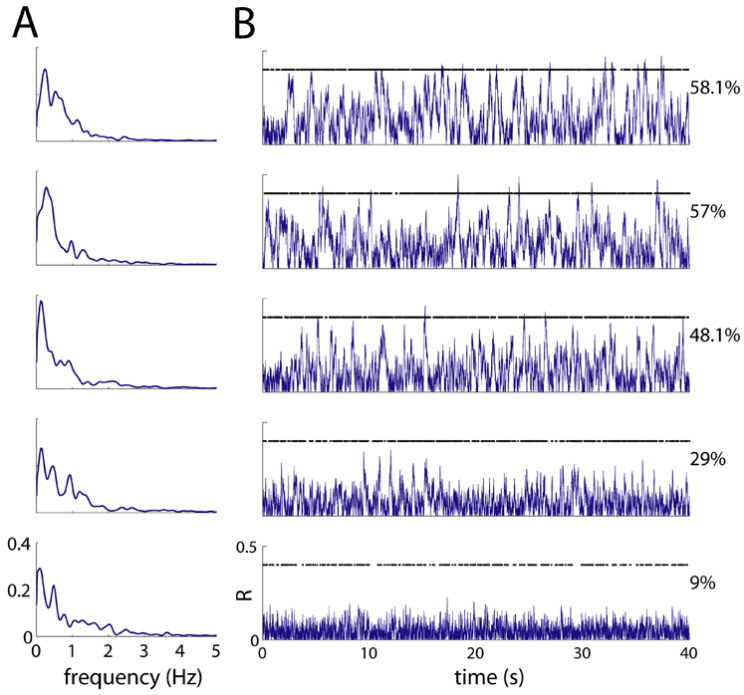
Coherent behavior and network attractors. A: Power spectral density of the dominant modes shown in figure 4 in the same order. The dominant modes are clearly oscillatory with at least one preferred frequency. The superposition of the oscillatory modes endows the spontaneous activity with coherent behavior in space and time. B: The normalized projection, *R* of the chosen modes onto the spontaneous activity yields the instantaneous contribution of each mode. Thus, the spontaneous activity can also be regarded as fluctuations of the network state around the basins of attraction of different attractors (modes). The black dots represent an instantaneous incursion into the basin of attraction of the corresponding mode. The percentage indicates the relative amount of time spent in the corresponding basin of attraction, i.e. the attractor's dwell time (see *Materials and Methods*).

As shown in *Materials and Methods*, the spontaneous network activity can be expressed as the summation of dominant patterns (modes) whose amplitudes are modulated in time. This modulation is represented by the traces in figure 4 on the right, which are mathematically obtained by projecting the spontaneous activity onto each dominant pattern (see *Materials and Methods*). The oscillatory nature of these traces is quantified in their power spectral density (Fig. 5A). Although they all fluctuate more strongly in the low frequencies, different modes do not necessarily oscillate at the same frequency. The fact that the modes are spatially extensive and that their temporal modulation is rather regular but not constrained to a specific time scale explains the emergence of coherent, complex dynamics in the neural network. In effect, the patchy structure of the dominant patterns in figure 4 means that segregated regions fluctuate coherently in time. In addition, these fluctuations occur in different time scales for different modes. Thus, the superposition of all these modes modulated in time results in the complex spatiotemporal patterns of the spontaneous activity, as recently observed in experimental data [1], [4], [8], [10].

According to the decomposition of spontaneous activity in modes, one expects that at each point in time a given mode prevails over the rest, i.e. at some points in time the spontaneous activity will mostly resemble one of the dominant spatial patterns. To test this prediction, one can use a “template matching” algorithm, similar to the strategy used to identify repetitive motifs in cortical songs [8]. The template in our case is one of the dominant patterns, which is compared with each snapshot of the spontaneous activity. Interestingly, this approach allows us to demonstrate the equivalence of the concept of dominant pattern and the concept of network attractor. In effect, one can think of the spontaneous activity as a series of random transitions between attractors of the stochastic network dynamics. To show that the dominant modes are actually these attractors we estimate their *basin of attraction* by applying the aforementioned template matching algorithm in the following way: we first calculate the correlation coefficient of the dominant patterns with each snapshot (see *Materials and Methods*). In other words, we calculate the instantaneous similarity between the spontaneous activity and its dominant patterns. We then check whether it is significant and if so, we mark that point in time with a black dot. This allows us to visualize when the state of the network approaches a given attractor (Fig. 5B) and to quantify the fraction of points in time when this happens. As expected, the dominant modes have “larger” basins, i.e. stronger attraction, the larger their eigenvalues are. In fact, the correlation coefficient between the eigenvalues and the fraction of time spent in the basin of attraction is *r* = 0.968. This demonstrates the equivalence between the concept of principal component or dominant pattern of the spontaneous activity and the concept of network attractor.

## Discussion

Using a general firing-rate model of neural dynamics [19], [20], we have shown how the network architecture determines the dominant patterns of the spontaneous activity. In particular, we have described mathematically the relationship between the connectivity matrix and the principal components of the spontaneous activity. The examples provided reveal how regularities in the connections lead to spatial patterns that vary in time but tend to reappear consistently. In the simulations described, these patterns contain several *modules* of excited and inhibited domains, characteristic of modular architecture and spontaneous activity in the cortex [5], [6]. The modules are functional rather than anatomical, since the domains wax and wane in time.

The relationship between network activity and network architecture has been extensively studied recently by several authors, but mostly at a macroscopic level, describing interactions between brain areas, or in a more general context of complex network architectures [11]–[16], [21]. Here, in contrast, we have focused on the microscopic level describing interactions of local neural groups, in particular, across a few cortical columns. The mathematical framework used in this article is similar to those used by other authors investigating complex biological networks [11], [12], [21], [22]. More specifically, we describe network interactions in terms of linear stochastic processes along the lines of such studies. In this manuscript, however, we have devoted special attention to the principal components of the spontaneous activity and we have demonstrated that they represent the basins of attraction of its stochastic dynamics.

Other groups have previously shown that the complexity of the architecture determines the complexity of the interactions between different brain areas [11], [12]. In particular, dense local connections and sparse long-range connections tend to generate large-scale, complex behavior [11], [12]. Moreover, the analysis of large-scale neuroanatomical data sets has revealed characteristic building blocks of the network architecture [13]. Combining those findings with the model presented here, it would be interesting to investigate the extent to which the dominant patterns of large-scale, spontaneous activity actually represent structural building blocks.

Here we have shown how to predict dominant patterns in the spontaneous activity from the network connections. From an experimentalist's perspective the inverse problem may be even more relevant, i.e., whether the network architecture can be reconstructed from the principal components of the spontaneous activity. The connectivity can be *inferred* from the spontaneous activity but not exactly determined. The limitations for this are twofold: technical and theoretical. The technical difficulties are due to the finite size of the data that allow us to calculate only a few principal components reliably. But even if infinite data sets were feasible, there is a fundamental limitation to perfectly retrieve the connectivity matrix. Equation (5) in *Materials and Methods* shows the relationship between the network architecture (implicit in *A*) and the covariance matrix of the spontaneous activity *C*, from which the dominant patterns are calculated. Whereas equation (5) is linear if we take the elements of *C* as the unknowns and the elements of *A* and *Q* as parameters, equation (5) is nonlinear if we take the elements of *A* as unknowns and the elements of *C* and *Q* as parameters. Due to the nonlinearity, when solving for *A*, the solution will not be unique in general. In fact, this is a well-known result of stochastic theory: the drift matrix (in our case, the connectivity matrix A) of a linear Langevin process (in our case, the spontaneous activity) cannot be uniquely retrieved from its covariance matrix [23], [24]. Nonetheless, an elegant way of overcoming this limitation has been recently proposed in the context of metabolomic networks [21]: first, some entries of the connectivity matrix are set as fixed parameters and then, a parametric solution is found. However, whereas this trick works efficiently for small networks, it becomes intractable for relatively large ones. Despite all these limitations, our results show that the connectivity of the network can indeed be qualitatively inferred. In effect, by looking at the modular structure of the dominant patterns one gets an idea of the connectivity kernel. For example, the spots of center surround inhibition in the dominant patterns of figure 4 have the size of the central wiggle in figure 1B.

In addition to the resolving power of our model to elucidate underlying anatomical connectivity from the patterns of spontaneous activity, it also provides an interpretation of the role of that activity in framework of neural network dynamics. The concept of attractor network has dominated computational neuroscience for about three decades [23], [25]–[29]. The idea that neural dynamics encode and store representations of stimuli in the form of attractors of the network dynamics has been fueled by the findings of several experimental studies in different systems [7], [30]–[33]. Moreover, it has been proposed recently that the highly consistent, spontaneously-evoked network up-states observed in cortical slices represent circuit attractors [1], [4]. Here, we have demonstrated that the principal components of the spontaneous activity can be interpreted as attractors of the stochastic background activity of the network. In particular, the eigenvalues of the covariance matrix roughly represent the fraction of time spent by the network in the basin of attraction (dwell time) of the associated eigenvector or principal component. Furthermore, changes in the principal component of the spontaneous activity during behavioral experiments can be used to quantify changes in the network connectivity and hence, to uncover Hebbian memory traces, as recently shown in an insect's brain *in vivo* [7].

It is worth mentioning that each spatial pattern (snapshot) of the spontaneous activity is not necessarily identical to any of the principal components. From a mathematical point of view, however, each spatial pattern can be expressed as a linear combination of principal components, provided that they are not degenerate, i.e. if all eigenvalues are different. Degeneracy appears when the connectivity matrix is “highly symmetrical”. For example, several sets of degenerate eigenvalues occur from a kernel like that in figure 1, which has rotational symmetry. By construction, the network also displays translation invariance (due in part to wrap-around boundaries). In reality, however, synaptic connections display large variability over the main architectural theme (see e.g., figure 3 in [34]). We have modeled this variability as 25% random connections on top of the architecture obtained from the Gabor kernel (see *Materials and Methods*). This is more than sufficient to remove degeneracy completely, as shown in Fig. 3C, where no eigenvalues coincide on the same spot. In principle, since the set of non-degenerate patterns forms a basis of the “snapshots space”, any arbitrary spatial pattern might be possible at any given time during the spontaneous activity. However, as mentioned in the previous paragraph, the spontaneous activity is biased to the dominant patterns proportionally to their eigenvalues and not any arbitrary pattern will realize. This implies that the repertoire or “alphabet” with which the network can encode and store information is constrained by the dominant modes, or equivalently, constrained by the network architecture.

One can possibly argue that any other basis different from the basis of principal components may be used to decompose the spontaneous activity. One may also wonder whether those alternative basis vectors could also be considered as attractors. In principle, one could choose another basis to decompose the spontaneous activity, but it would not be adequate in the sense that it would not make the relationship between the basis and the network architecture explicit, as it is the case with the basis of principal components. To demonstrate this, we have also used a Fourier basis (see figures in *Supporting Information*). In particular, we have first calculated the power spectrum of the spatial frequencies for each snapshot of the spontaneous activity. Then, we have averaged the power spectra of all snapshots. Interestingly, the averaged spectrum clearly reveals an elevated ring (see figure S1), whose radius roughly corresponds to the reciprocal of the period of the Gabor kernel, indicating that the Fourier decomposition captures an important feature of the network connectivity. We note that the ring does not have a uniform height. This means that we can rank the dominant spatial frequencies along the ring according their power. In figure S2, we display the spatial patterns associated with the six dominant spatial frequencies. However, as seen in figure S3, the dominant patterns of the Fourier basis cannot be regarded as attractors because their instantaneous correlation with the spontaneous activity is negligible. In other words, the projections of those vectors onto the spontaneous activity do not quantify the dwell time, which is the idea behind the concept of attractor. In fact, as seen in figure S3, the network spends 0% of the time in the basins of the Fourier modes.

Here we have modeled the spontaneous activity as a linearly stable, stochastic system. The assumption of linearity comes from the observation that the baseline firing rates of neurons in the absence of stimulation are typically much lower than during stimulation [7] and far from saturation. In such stochastic systems (like eq. 3), the stability criterion is that all eigenvalues of the linear operator (*A*, in eq. 3) has magnitude less than one. If one or several modes have eigenvalues which do not fulfill this condition, the spontaneous activity will grow quickly in time entering the saturation regime (nonlinearity in equation 1). This kind of behavior resembles an epileptic seizure and the physiological mechanisms leading to this instability can be studied to some extent with our approach. For example, an obvious way of inducing a seizure in the network consists in reducing the relaxation parameter, α until one mode of *A* becomes linearly unstable. Interestingly, some *in vitro* preparations of the cortex display distinct episodes of spontaneous network activity [35]. Thus, in real networks, the parameter α is not strictly constant but varies in time, although at a slower time scale than that of the activity fluctuations. In addition, the dimensionality of the dynamics, i.e. the number of modes that significantly contribute to the spontaneous activity, increases close to a transition between episodes [35]. These transitions (or bifurcations) are associated with changes of neuronal excitability, which are originated in ion shifts from inside to outside of the neurons and in oxygen limitations in the brain tissue [35], [36].

In this paper we have exclusively focused on the spontaneous activity of neural networks. At this point, however, we should say a few words about the behavior of the model in response to stimulation. In the model used here, a stimulus will drive equation (1) into saturation of firing rates quickly. This happens because in various neurons the inhibition and the relaxation rate cannot catch up with the excitation plus the stimulus drive. The spatial patterns of saturated firing rates will generally depend on which neurons are driven and how much (*I_i_*, in equation 1). In particular, since the dominant modes are built-in in the network architecture, a spatial input pattern *I_i_* may *resonate* with the dominant mode that it most resembles until reaching saturation. Using the jargon of synergetics [23], the whole network activity will then be *enslaved* by that dominant mode.

## Materials and Methods

### Mathematical model of spontaneous neural activity

We start with a general model of neural network dynamics of the Wilson-Cowan type [19], [20] that describes the variations of firing rate in the neurons due to synaptic and external currents. The model is slightly modified to take into account the effect of intrinsic noise:
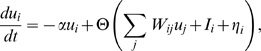
(1)where *u_i_* represents the activity (firing-rate) of the *i*-th neuron in the network; α is the inverse of the relaxation time; *W_ij_* is the synaptic strength between neuron *i* and *j*, i.e. the connectivity matrix; *I_i_* is the external input to the *i*-th neuron; and η*_i_* is a random, fluctuating input into the *i*-th neuron (modeled as white noise) accounting for channel noise, spontaneous synaptic release and other sources of biophysical variability. The nonlinear function Θ(*x*), typically a sigmoid of hyperbolic-tangent type, limits the growth of its argument to an asymptotic value which accounts for the saturation of firing rates in real neurons.

In the absence of stimulation, the external inputs to all neurons in the network vanish, *I_i_* = 0. Thus, the only driving force are intrinsic, random fluctuations η*_i_* with standard deviation σ. Because those fluctuations are not sufficiently strong to evoke large variations of the firing rate, the saturation due to the nonlinear function Θ can be ignored in the study of spontaneous activity. In these conditions, the dynamical equations can be easily linearized around the quiescent state of the network:
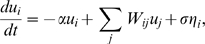
(2)where we have factorized the last term into σ and η*_i_*, which now has unitary standard deviation. In practice, to simulate system (2) the differential equations are discretized in time with a finite time step Δ*t*, taking the form
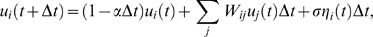
which in vector notation can be rewritten as
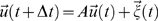
(3)using 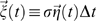, and *A*≡(1−αΔ*t*) *E*+*W*Δ*t*, where *E*≡δ*_ij_* is the identity matrix.

### Estimation of dominant patterns from the traces of spontaneous activity

The dominant patterns can be directly computed from the time series of spontaneous neural activity obtained either from a model, like the one exposed above or from actual recordings of neural activity. To this end, one first calculates the covariance matrix of the neural traces *u_i_*(*t*)
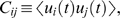
where the brackets indicate temporal average. Equivalently, in vector notation the covariance matrix of the spontaneous activity reads
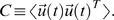
(4)


Then, one calculates the eigenvectors and eigenvalues of *C*. The covariance matrix *C* is symmetric by construction and therefore, it has only real eigenvectors and eigenvalues. The eigenvectors of *C* are by definition the principal components of the spontaneous activity, i.e. the dominant patterns or modes, and they represent the spatial patterns in which it can be decomposed. In other words, each snapshot of the spontaneous activity can be represented as a linear superposition of these modes, being their relative weights different in each snapshot.

The overall weight of each mode in the spontaneous activity is given by the magnitude of the associated eigenvalue. This poses an interesting link between the dominant patterns and the concept of attractor in neural dynamics, as recently illustrated in the olfactory system of an insect [7], [32]. In effect, it can be shown that the temporal average of the similarity (projection) of the spontaneous patterns onto the dominant eigenvector of the covariance matrix, or first principal component, is maximal. In other words, the neural activity fluctuates most of the time around the *basin of attraction* of the dominant pattern. The amount of time spent around the basin of attraction of the remaining principal components is proportional to the magnitude of their eigenvalues.

### Mathematical proof of the relationship between connectivity and dominant patterns

We start with equation (3); multiplying it with its transpose and averaging in time we obtain
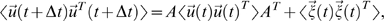
where all terms containing a single product with noise, 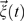 have vanished after averaging. As in (4) we now define the covariance matrix of the spontaneous activity as
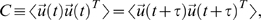
where the equality is justified by the stationary character of the spontaneous activity, i.e. temporal averages are invariant under translation in time. Note that *C* is now calculated directly from the model rather than being estimated from the traces of simulated neural activity. Then, defining *Q* as the covariance matrix of the intrinsic noise
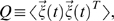
we arrive at a matrix equation relating the covariance matrices with the network connectivity via *A*

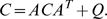
(5)


Although our derivation is valid for any *Q*, we can assume for our purposes that the noise sources are uncorrelated in different neurons, i.e. *Q* = (σΔ*t*)^2^ *E*. Note that in this case, *Q* is a diagonal, constant matrix.

Our goal now is to solve for *C* in (5). To this end, we start considering the eigen-decomposition of matrix *A*

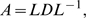
(6)where the columns of matrix *L* are the eigenvectors of *A* and the diagonal matrix *D* contains the associated eigenvalues, λ. Note that the eigenvalues of *A* are the eigenvalues of *W* scaled by the factor Δ*t* and shifted by the constant 1−αΔ*t*. In a purely deterministic system, i.e. in the case of ξ*_i_*(*t*) = 0, the stability of the model with respect to any finite input pattern *I_i_*(*t*)≠0 is guaranteed if all eigenvalues of *A* are negative, which can always be achieved if α is sufficiently large. However, in the case of the stochastic system (3) considered here, stability is guaranteed if all the eigenvalues of *A* have magnitude less than unity [37], [38], which means that the spontaneous activity cannot grow infinitely in response to noise, but will remain fluctuating around its mean value.

Since the connectivity matrix *W* is not symmetric in general, neither is *A*, and therefore, *A* can have complex eigenvalues and eigenvectors. As a result we need to replace the transpose operation *X^T^* by the conjugate transpose operation *X* ^†^. Then, substituting (6) in (5) we obtain
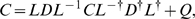
(7)where we have used *L* ^−†^≡(*L* ^−1^)^†^ = (*L* ^†^)^−1^. Multiplying (7) by *L* from the left, then by *L* ^†^ from the right and defining 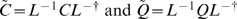 we get
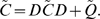
(8)


Since *D* is a diagonal matrix, equation (8) can easily be rewritten in terms of the matrix components
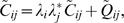
where the asterisk denotes complex conjugation. Then, solving for 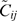 one has
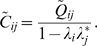
(9)


Multiplying (9) by *L* from the left and by *L* ^†^ from the right we arrive at the most relevant theoretical result of this paper: the covariance matrix of the spontaneous activity is determined by the covariance matrix of the intrinsic noise and the eigenvalues of the connectivity matrix:
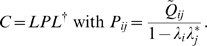
(10)


Finally, taking into account that the spatial modes of the spontaneous activity are the eigenvectors of *C*, we calculate the eigen-decomposition of the covariance matrix
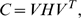
(11)where the columns of *V* are the eigenvectors and the diagonal matrix *H* contains the corresponding eigenvalues (since *C* is symmetric, all eigenvalues and eigenvectors are real and in addition, *V* is an orthogonal matrix, i.e. *V* ^−1^ = *V^T^*). The absolute value of the eigenvalues indicates the relative importance of the corresponding eigenvectors in the spontaneous activity of the network.

Summing up, the modes of the spontaneous activity are fully determined by the connectivity matrix via the eigenvectors and eigenvalues of matrix *A*. This theoretical derivation allows us to compare the dominant patterns obtained from the covariance matrix of the time series *u_i_*(*t*), via (4), with the dominant patterns obtained from the covariance matrix derived from the connectivity matrix via (10) and (11). As shown in *Results*, there is a remarkable agreement between the results of both methods.

### Decomposition of the spontaneous activity in dominant modes

Let 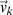 be the *k*-th eigenvector of the covariance matrix, i.e. the *k*-th dominant pattern or mode. The spontaneous activity 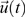 can be expressed as a linear combination of these modes, which are pairwise orthonormal, i.e. 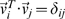:
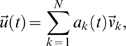
(12)where *N* is the number of modes (which coincides with the number of neurons in the network) the coefficients *a_k_*(*t*) represent the instantaneous contribution of each mode to the spontaneous activity and are obtained using 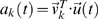. The eigenvalues λ*_k_* associated with the dominant modes are the mean squared amplitude, i.e. the variance, of these coefficients 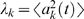. Therefore, only the modes with larger eigenvalues are relevant in practice for expansion (12). The decomposition of the spontaneous activity in dominant modes is analogue to the decomposition of the vibrations of a drum's membrane in vibrating modes, i.e. cylindrical harmonics (Bessel functions). In general, the decomposition of spatiotemporal neural activity into modes has been used by other authors in different contexts [35], [39].

### Dominant modes and network attractors

As mentioned above, the dominant patterns of the spontaneous activity can be regarded as attractors of the network dynamics. A link between both concepts is provided by the estimation of their basins of attraction. To this end, we first calculate the normalized projection of the principal components on each snapshot of the spontaneous activity (i.e. the correlation coefficient, *r*(*t*)). The magnitude of this correlation is plotted in figure 4. Specifically, if the *k*-th principal component is 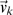, *r*(*t*) is given by
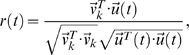
and we plot *R*(*t*) = |*r*(*t*)|. We then calculate the confidence interval using Fisher's z-transformation:
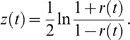



The confidence interval of the random variable *z* at each point in time is given by 
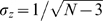
, where *N* is the dimension of the vectors 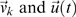, i.e., the number of neurons. Thus, the fraction of *z*(*t*) samples exceeding σ*_z_* in absolute value is a measure of the attraction of the spontaneous activity to the *k*-th principal component, i.e. a measure of its basin of attraction. For visualization purposes, in figure 5B we show the fraction of *z*(*t*) samples exceeding 3σ*_z_* in absolute value (black dots).

### Numerical details of simulations and analyses

All simulations and analyses were performed in Matlab 6.5. The model consisted of a square network of 30×30 = 900 units (neurons). The elements of the connectivity matrix *W_ij_* were first obtained by convolving the kernel in Fig. 1a with a delta function of unitary amplitude on the *i*-th neuron of the network and considering periodic (wrap around) boundary conditions. Then, a random connectivity matrix of normally distributed synaptic weights was added to introduce 25% variability. Finally, the elements were divided by the norm of the matrix and multiplied by 1.03. This, together with the following parameter choices ensured the stability of stochastic system (3): α = 1, σ = 1, Δ*t* = 0.2.

Our model is adimensional in nature. In order to endow the model with biological spatiotemporal scales we first notice that the period of the Gabor kernel must be within the range of 400 to 900 µm [17]. We take the period to be 0.7 mm. Then, we observe that 10 s of spontaneous cortical traces embrace 6 to 8 peaks of fluctuating activity [6]. This implies that the units of our integration time step Δ*t* must be s×10^−2^.

Figure 3D displays the correlation between the predicted and the observed dominants patterns. More technically, if 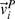 is the *i*-th predicted pattern and 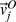 is the *j*-th observed pattern, then the correlation matrix *R_ij_* is defined as:
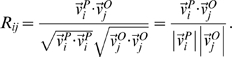



If the predicted and the observed patterns are similar, one expects larger correlation values along the diagonal of the matrix, as seen in figure 3D.

## Supporting Information

Figure S1 Power spectrum of the spatial frequencies (Fourier decomposition) averaged across all snapshots of the spontaneous activity. The dominant Fourier modes are arranged along a ring of inhomogeneous height, which indicates the relative weight of each mode in the stochastic network dynamics. The radius of the ring corresponds to the reciprocal of the period of the oscillation in the Gabor kernel.
(1.13 MB TIF)

Figure S2 Spatial patterns with largest power in the Fourier decomposition of the spontaneous activity. The patterns exhibit the spatial frequency of the Gabor kernel. Thus, the dominant Fourier modes capture a relevant feature of the network architecture.
(1.70 MB TIF)

Figure S3 Normalized projection of the dominant Fourier modes onto the spontaneous activity. The projections are negligible, which means that, contrary to the principal components, the Fourier modes cannot be considered as attractors of the stochastic network dynamics.
(0.41 MB TIF)

Movie S1 Simulated Spontaneous Activity. The spontaneous activity organizes in complex spatiotemporal patterns (some snapshots of the movie are also shown in figure 2). The color scale indicates spontaneous firing rates in arbitrary units: red being above firing baseline (represented in green) and blue below. The spontaneous patterns reveal some spatial modularity, reminiscent of the spatial patterns observed via voltage sensitive dye imaging in V1 of cats. In effect, red and blue areas segregate forming domains of approximately the same extension. These modules vary in time but have a pronounced tendency to reemerge. Black scale bar: 0.7 mm.
(9.89 MB MPG)
