# Correction: On How Network Architecture Determines the Dominant Patterns of Spontaneous Neural Activity

**DOI:** 10.1371/annotation/2c9bfbcb-6b96-4d77-bfe3-10c5988150b8

**Published:** 2008-05-15

**Authors:** Roberto F. Galán

There was an error in the author's name in the copyright statement of the article. His name should appear as Roberto Fernández Galán.

