# Supplementary figures and images for: On How Network Architecture Determines the Dominant Patterns of Spontaneous Neural Activity
Source: PLoS One. 2008 May 14;3(5):e2148. doi: 10.1371/journal.pone.0002148 (PMC2374893; doi:10.1371/journal.pone.0002148)

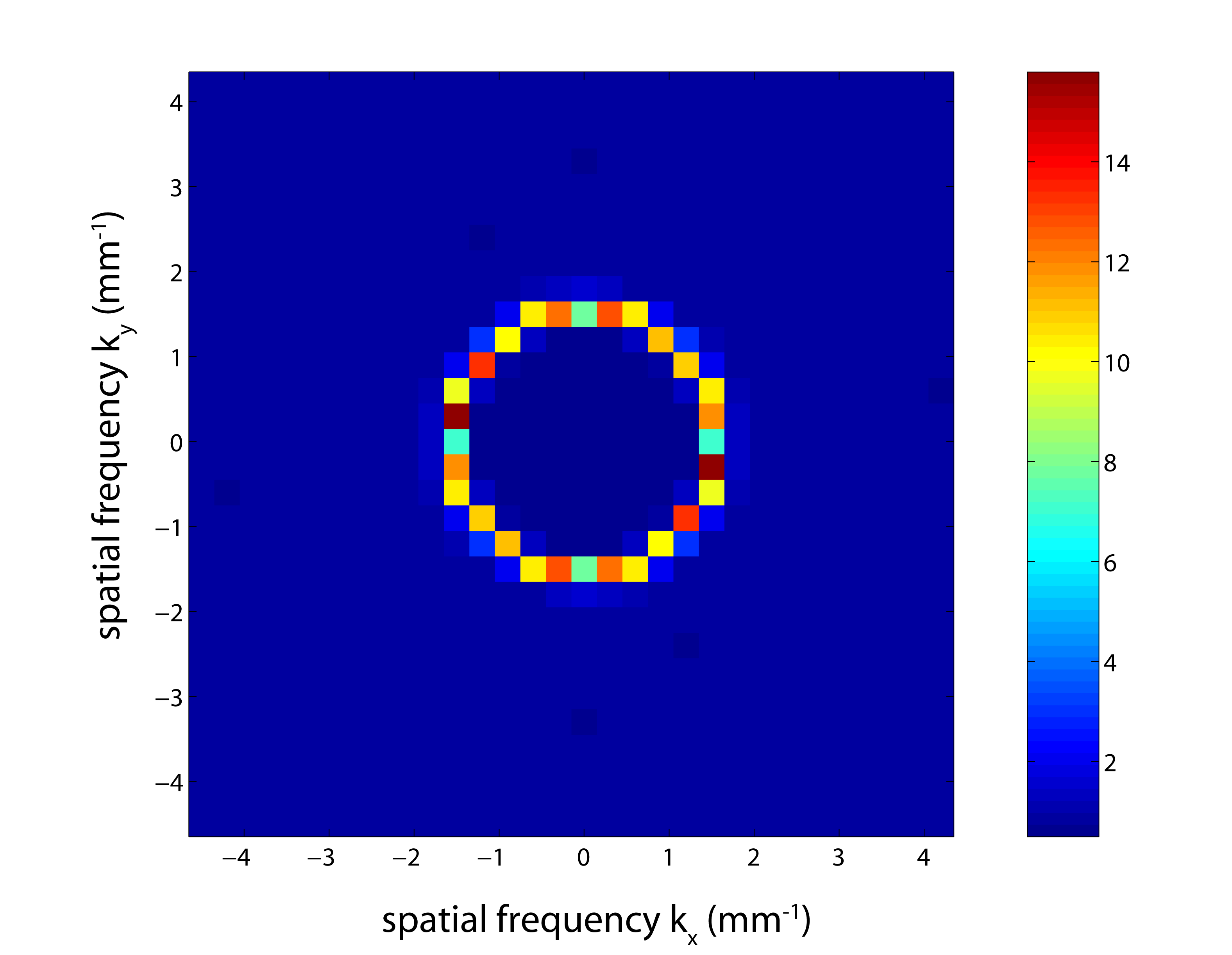

Supplement: Figure S1 — Power spectrum of the spatial frequencies (Fourier decomposition) averaged across all snapshots of the spontaneous activity. The dominant Fourier modes are arranged along a ring of inhomogeneous height, which indicates the relative weight of each mode in the stochastic network dynamics. The radius of the ring corresponds to the reciprocal of the period of the oscillation in the Gabor kernel. (1.13 MB TIF) [file pone.0002148.s001.tif]

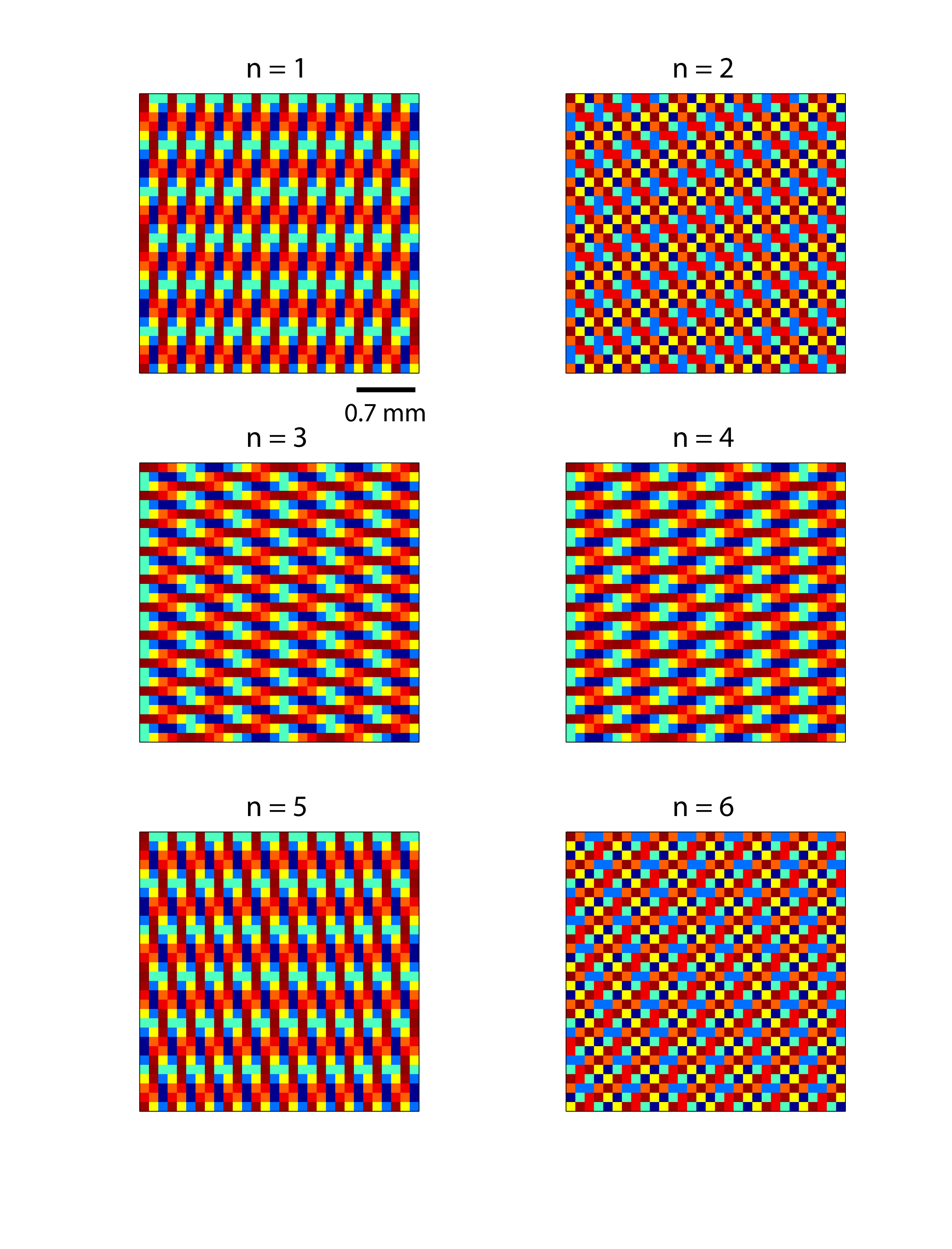

Supplement: Figure S2 — Spatial patterns with largest power in the Fourier decomposition of the spontaneous activity. The patterns exhibit the spatial frequency of the Gabor kernel. Thus, the dominant Fourier modes capture a relevant feature of the network architecture. (1.70 MB TIF) [file pone.0002148.s002.tif]

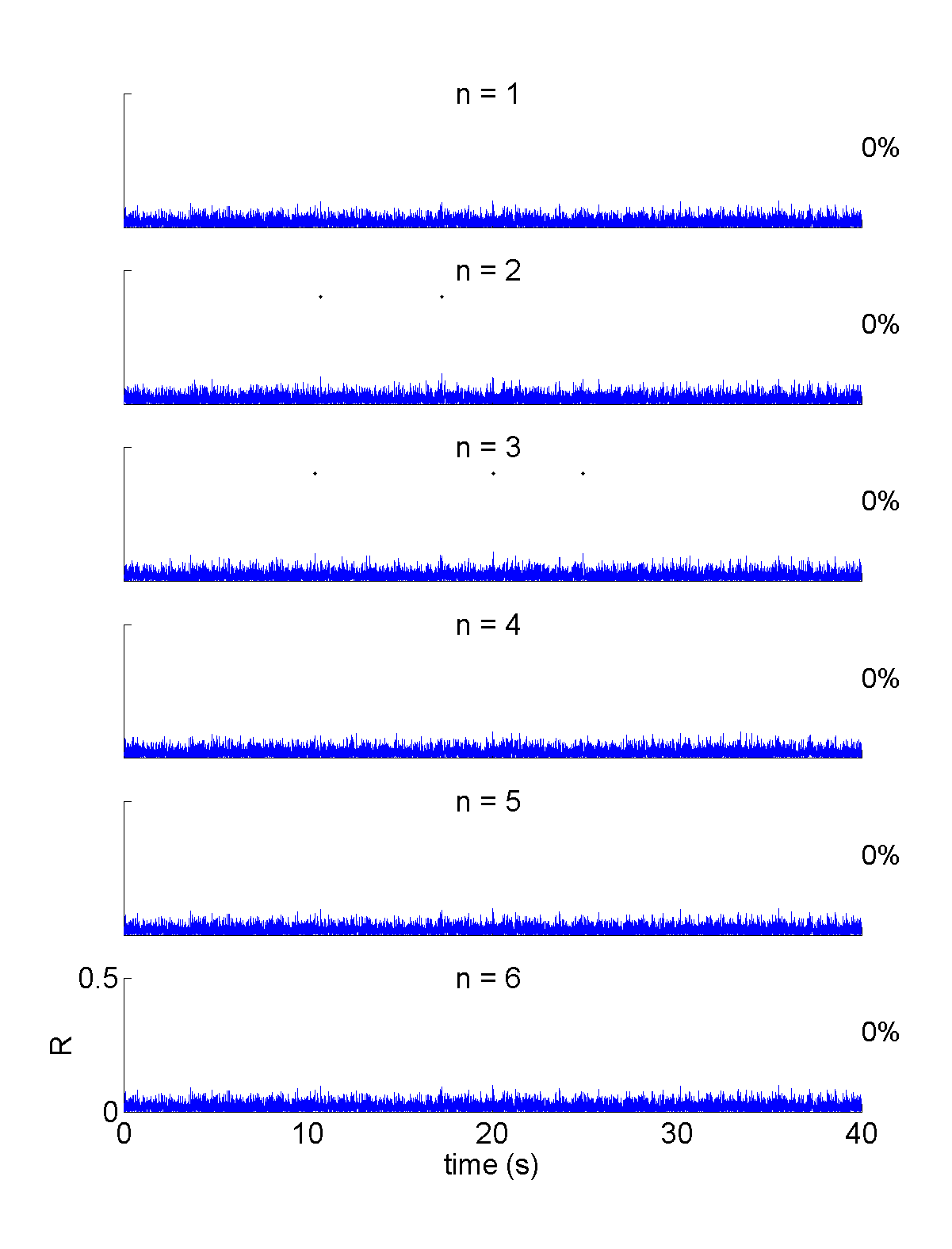

Supplement: Figure S3 — Normalized projection of the dominant Fourier modes onto the spontaneous activity. The projections are negligible, which means that, contrary to the principal components, the Fourier modes cannot be considered as attractors of the stochastic network dynamics. (0.41 MB TIF) [file pone.0002148.s003.tif]
